# Relationship of Early Anemia with Neurodevelopment and Brain Injury in Very Low Birth Weight Preterm Infants—A Prospective Cohort Study

**DOI:** 10.3390/nu14224931

**Published:** 2022-11-21

**Authors:** Xiaotong Wang, Jiajia Jing, Saijun Huang, Xiaoying He, Pingming Gao, Hailin Li, Zongyu Lin, Per Torp Sangild, Yanna Zhu

**Affiliations:** 1 Department of Maternal and Child Health, School of Public Health, Sun Yat-sen University, Guangzhou 510080, China; 2 Department of Child Health Care, Foshan Woman and Children’s Hospital, Foshan 528000, China; 3 Department of Pediatrics, Foshan Woman and Children’s Hospital, Foshan 528000, China; 4 Department of Pediatrics and Adolescent Medicine, Rigshospitalet, DK-1870 Copenhagen, Denmark

**Keywords:** anemia, very-low-birth-weight infants, preterm, neurodevelopment

## Abstract

Anemia is associated with neurodevelopmental delays and brain injury in infants and toddlers, but whether early anemia has a similar effect in neonatal preterm infants is largely unknown. Thus, this study aimed to determine the relationship of early anemia with neurodevelopment and brain injury in very-low-birth-weight (VLBW) preterm infants within the neonatal period. A prospective cohort study including 110 VLBW preterm infants was conducted in Southern China from 2016 to 2018. All participants were followed from birth to 1 month corrected age. Early anemia is defined as hemoglobin of ≤145 g/L within the first week after birth. The non-anemic group (control group, N = 55) was 1:1 matched with the early anemia group (N = 55) according to birth weight and gestational age. Neurodevelopment at 1 month corrected age and brain injury within 1 month corrected age were measured by neonatal behavioral neurological assessments (NBNA) and cranial ultrasound, respectively. Compared to the control group, the early anemia group had a lower score in behavioral ability in the NBNA test [11 (10–12) vs. 10 (9.5–11), *p* = 0.033]. Early anemia was negatively associated with the NBNA total score (β= −0.680, 95% CI: −1.300, −0.059), especially with the behavioral ability score (β= −0.504, 95% CI: −0.941, −0.067) after adjusting for the confounders. However, no association between early anemia and brain injury was observed. In conclusion, in VLBW preterm infants, early anemia is negatively correlated with neurodevelopment, especially with behavioral ability.

## 1. Introduction

Preterm infants account for 10% of newborn births annually [1]. Preterm infants, especially those with very low birth weight (VLBW), have a higher risk of anemia than full-term infants due to immature bone marrow hematopoietic function [2,3]. The diagnostic criteria for anemia in preterm infants under 6 months of age are different across the world [4]. In China, the standard of early anemia is hemoglobin (Hb) ≤ 145 g/L within the first week after birth [5]. There are 38.1~41.2% of preterm infants that suffer from anemia during hospitalization in China [6].

It is noteworthy that anemia affects preterm infants’ brain development, causing neurodevelopmental delays and brain injury [7]. Neurodevelopment refers to different developmental domains based on different tools, such as motor development, behavioral ability, and communication. Studies found that severe neonatal anemia was associated with poorer long-term neurodevelopment in preterm infants, especially in those with low-birth-weight [7,8,9,10]. In addition, lower Hb level was correlated with poor gross motor development of preterm infants at the adjusted age of 30 months [7,11]. However, previous studies mostly focused on the relationship of anemia with neurodevelopment at 2 years old or older [12,13], and limited evidence on the neurodevelopment outcome during the neonatal period [14]. What is more, severe anemia at birth (Hb < 70 g/L) was associated with impaired brain white matter in MRI tests [13,15]. Nevertheless, most research has focused only on severe anemia, and studies of non-severe (70 g/L ≤ Hb < 145 g/L) early anemia are lacking. Thus, it is necessary to explore the relationship of early anemia (70 g/L ≤ Hb < 145 g/L) with neurodevelopment and brain injury during the neonatal period in VLBW preterm infants.

To sum up, based on the diagnostic criteria of early anemia in China (Hb ≤ 145 g/L), this study reported the changes in Hb concentration in VLBW preterm infants during the first 28 days after birth. This study aimed to explore the relationship between early anemia and neurodevelopment and brain injury in VLBW preterm infants.

## 2. Materials and Methods

### 2.1. Study Design

This prospective cohort study was conducted in the neonatal intensive care unit of Foshan Woman and Children’s Hospital, Guangdong Province, Southern China, from 2016 to 2018. The participants were recruited at birth and were followed up at 1 month corrected age. The Ethics Committee of Sun Yat-sen University approved this study and written informed consent was obtained from parents. This study was conducted in accordance with the Declaration of Helsinki.

### 2.2. Participants

The inclusion criteria were the following: transferred to neonatal intensive care unit within 24 h after birth, birth weight less than 1500 g, had Hb measurement during the first 28 days after birth, singleton or multiple births, and willing to join the study. Infants with major congenital abnormalities, congenital metabolic diseases, or Hb concentrations ≤ 70 g/L were excluded. The study followed up from birth to 1 month corrected age. There were 55 participants recruited in the early anemia group. The control group included 55 non-anemic VLBW infants, which were matched with the early anemia group according to gestational age and birth weight.

### 2.3. Data Collection

At baseline, the Hb concentrations of the participants were collected from clinical records at least once a week from birth to the first 28 days after birth. Hematology tests were conducted depending on the participants’ clinical needs. Baseline characteristics of infants in the early anemia group and the control group including maternal age (years), delivery mode (cesarean section/spontaneous labor), gestational age (weeks), biological sex, birth weight (Kg), birth head circumference (cm), and birth length (cm) were derived from clinical records. Moreover, nutrition supply (introduction and duration of parenteral and enteral feeding (day of life and days)) and respiratory condition (days on intubated ventilatory, non-invasive ventilator, and extra oxygen support) were also collected from clinical records. During the follow-up study, the outcomes were measured by trained inspectors.

### 2.4. Exposure and Outcome Variables

Early anemia was defined as a Hb of ≤145 g/L detected at any time within the first week of birth. In this study, the early anemia group included infants with mild to moderate anemia (70 g/L ≤ Hb levels ≤ 145 g/L) [5].

Outcome variables included neurodevelopment and brain injury. Neurodevelopment was evaluated by the neonatal behavioral neurological assessment (NBNA) test within 1 month corrected age. Bao Xiulan and others developed NBNA based on Brazelton’s Newborn Behavior Assessment Scale [16]. The stability and reliability of NBNA has been confirmed in Chinese infants [17]. This test is a comprehensive evaluation system for evaluating neonatal neurological function and can reflect the overall functional state of the brain. NBNA is composed of five parts: behavioral ability, passive muscle tone, active muscle tone, original reflex, and general assessment. Each part contained 6, 4, 4, 3, and 3 items, respectively. For each item, it can be scored on three levels (0, 1, 2). The total NBNA score is the sum of the five parts mentioned above with a maximum score of 40. Newborns with a score < 37 were usually considered abnormal and scores ≥ 37 were considered normal. The behavioral ability reflected infants’ adaption to external stimulation, such as light, sound, voice, and comfort [17]. It was different from other scales, such as the Bayley Scales of Infant Development and General Movements. All evaluation processes were carried out in the medical room by well-trained inspectors, and all the inspectors were blinded to the early anemia group.

Brain injury was measured by cranial ultrasound within 1 month corrected age, and the results were derived from clinical records. The results included intraventricular hemorrhage, ventricular cyst, and ventricular widening. Preterm infants lack specific clinical manifestations for the early stage of brain injury, and imaging and other auxiliary examinations are of great value in predicting and evaluating the severity of brain injury. Ultrasound examination is a non-invasive and convenient operation [18].

### 2.5. Statistical Analysis

Statistical analysis was performed using SPSS (v22, IBM, Chicago, IL, USA, 2013). Quantitative variables were presented with a mean (standard deviation) or median (interquartile ranges), as appropriate. Quantitative variables were presented with numbers (percentages). The scores of the NBNA test (behavioral ability, passive muscle tone, active muscle tone, original reflex, and general assessment) were treated as continuous variables. First, the changes in Hb concentration during 28 days after birth were described by a line chart, which contained error bars of the confidence interval (CI) and mean levels of Hb concentration of the two groups. Secondly, the Mann–Whitney U test and Chi-squared test were used to analyze the differences in baseline characteristics of infants in the early anemia group and the control group. Third, NBNA scores were analyzed by the Mann–Whitney U test. In addition, the generalized linear model was used to determine associations between early anemia and neurodevelopment, and brain injury. Univariate analyses were performed as model 1. Multivariable models adjusted for gestational age, biological sex, delivery mode, birth weight, birth head circumference, birth length, and nutrition supply. *p* < 0.05 was considered statistically significant.

## 3. Results

### 3.1. Baseline Characteristics in the Early Anemia Group and the Control Group

In all participants, the median gestational age was 30.43(29.29–32.29) weeks, the median birth weight was 1310(1160–1400) g, and the median birth head circumference was 27(26–28) cm. As shown in Table 1, compared to the control group, the early anemia group reached enteral feeding volumes of 120 mL·kg^−1^·d^−1^ later (*p* = 0.045). However, there were no significant differences in maternal factors (maternal age, gravidity, parity, cesarean section rate, and singleton rate), neonatal factors (gestational age, birth weight, birth head circumference, birth length, the distribution of sex), nutrition supply, or respiratory condition between the early anemia group and the control group (*p* > 0.05).

### 3.2. The Changes in Hb Concentration in VLBW Preterm Infants

**Figure 1:**
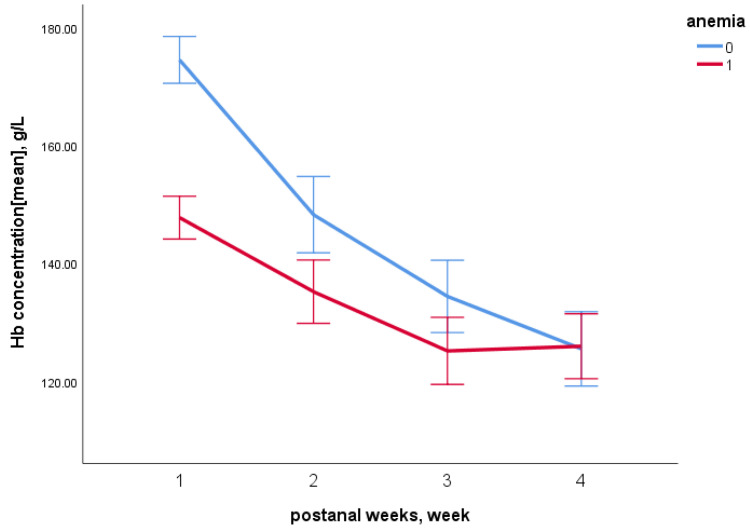
Changes of Hb concentration in the early anemia group and control group within 28 days after birth. Zero referred to the control group, and one referred to the early anemia group.

As shown in Figure 1, the average Hb concentration of the two groups decreased within 28 days after birth. The difference in Hb concentration between the early anemia group and the control group was greatest at birth but gradually narrowed during the first 28 days after birth. There was no significant difference in Hb concentration between the two groups on the 28th day.

### 3.3. The Relationship between Early Anemia and Neurodevelopment

Comparisons of NBNA scores between the early anemia group and the control group were shown in Table 2. In this study, 63.5% of participants in the control group had normal NBNA scores ( ≥ 37 scores), while 47.9% of those in the early anemia group reached normal levels. The infants in the early anemia group scored lower in behavioral ability in the NBNA test [11 (10–12) vs. 10 (9.5–11), *p* = 0.033] than those in the control group. However, no significant differences were observed in scores of passive muscle tone, active muscle tone, original reflex, general assessment, and NBNA total score between the two groups (*p* > 0.05).

The association between early anemia and neurodevelopment was presented in Table 3. In model 1, univariate analysis showed a negative correlation between early anemia and behavioral ability (β = −0.485, 95% confidence interval (CI): −0.890, −0.080). After adjusting for gestational age, sex, delivery mode, and birth weight in Model 2, early anemia was negatively correlated with NBNA total score (β = −0.650, 95% CI: −1.215, −0.085), especially with the behavioral ability (β = −0.606, 95% CI: −0.995, −0.218). Furthermore, in model 3, after adjusting the days of reached enteral feeding volumes of 120 mL·kg^−1^·d^−1^ based on Model 2, the association remained significant, that early anemia had a negative relationship with NBNA total score (β = −0.748, 95% CI: −1.36, −0.129), especially with the behavioral ability (β = −0.457, 95% CI: −0.840, −0.020). However, there was no significant association of early anemia with passive muscle tone, active muscle tone, original reflex, and general assessment in this study.

### 3.4. The Relationship between Early Anemia and Brain Injury

Comparison of the cranial ultrasound detection in the early anemia group and the control group is shown in Table 4. Compared with the control group, the early anemia group showed higher incidences of intraventricular hemorrhage (15.0% vs. 12.7%), ventricular cyst (22.5% vs. 10.9%), ventricular widening (20.0% vs. 9.1%), but the differences were not statistically significant (all *p* > 0.05).

## 4. Discussion

This prospective cohort study found that the average Hb concentration of VLBW preterm infants showed a downward trend within 28 days after birth. What is more, early anemia was negatively correlated with NBNA total score, especially with behavioral ability. However, no significant correlation between early anemia and brain injury was observed.

In the present study, the Hb concentrations of preterm infants with early anemia decreased continuously during the first 28 days after birth, which was consistent with other research [10,19]. The reason might be that in preterm infants, EPO production decreases, and EPO catabolism accelerates [20]. A low level of EPO persists throughout the postnatal period, resulting in lower Hb levels. Notably, in this study, the higher the initial level of Hb, the steeper the decrease was during the first 28 days after birth. The possible reason was that healthcare professionals were more concerned about preterm infants with lower initial levels of Hb than those with a higher initial level of Hb. The results suggested that it is also important to monitor Hb changes in infants with a normal level of Hb at the early stage of life.

The present study found that in VLBW preterm infants, early anemia (70 g/L < Hb levels ≤ 145 g/L) was negatively correlated with NBNA total score, especially with behavioral ability. The correlation between early anemia and neurodevelopment still existed after correcting the confounders. The results were partially in accordance with the previous studies. Previous studies have suggested that initial Hb levels < 75 g/L were an important factor for neurodevelopmental abnormalities in newborns [7,12]. The possible explanation for this association might be that the first 28 days after birth is an essential time for rapid brain growth and development of preterm infants born at less than 34 weeks of gestation [21], but anemia decreases the oxygen-carrying capacity of Hb to the cerebral blood flow and declined energy metabolism [22], causing the abnormal neurodevelopment outcomes. However, studies of the relationship between early anemia and neurodevelopment have used different scales, and the definition of early anemia varied, thus this makes it difficult to compare between studies. Even though the results of this study suggested that in this category of high-risk infants, the expected value of early Hb levels should be raised when assessing their risk for neurobehavioral abnormalities.

However, for the relationship between early anemia and brain injury, there was no correlation found in this study. This result was different from previous studies. In previous studies, Hb levels < 70 g/L were associated with impaired brain white matter in MRI tests, and the severity and type of neurodevelopmental consequences were essentially consistent with the extent and location of the impairment on MRI, both in full-term [13,23] and preterm infants [15,24]. The disparities in results may be explained by the fact that in this study anemia was not as severe as in other studies. Another possible reason was that in the present study, brain injury was detected by ultrasound, which was not as sensitive as MRI. Ultrasound examination has a lower risk of injury in preterm infants than MRI, thus it is a more routine choice in Chinese hospitals. However, the changes in the brain parenchyma and white matter could not be detected by ultrasound. Therefore, we could not directly deny the association between early anemia with Hb levels ≤ 145 g/L and brain injury. A systematic review [25] has suggested that increasing basal hemoglobin levels in preterm infants may reduce the incidence of brain injury such as intracranial hemorrhage in preterm infants, but these results are still controversial.

The strength of this study was that it highlighted the importance of early anemia in the health management of VLBW preterm infants. Unlike previous studies that mostly focused on severe anemia, this research focused on early anemia with 70 g/L < Hb ≤ 145 g/L. In this study, early anemia in VLBW preterm infants was mild to moderate anemia and did not reach the level of severe anemia. However, there was still a limitation of this study, which was a lack of diagnosis of brain injury from MRI. Although studies had shown strong concordance between cranial ultrasound diagnosis and MRI diagnosis, MRI diagnosis was mostly used in the same type of studies, thus more caution should be exercised when making cross-sectional comparisons.

## 5. Conclusions

To sum up, in VLBW preterm infants born at less than 34 weeks of gestation, the Hb levels showed a downward trend within 28 days after birth. Early anemia was negatively correlated with neurodevelopment, especially with behavioral ability in VLBW preterm infants. However, no significant association between early anemia with brain injury was observed. The current findings suggested that neurodevelopment in VLBW preterm infants with early anemia should be monitored beginning in the neonatal period, even if the anemia status did not reach a severe level.

## Figures and Tables

**Table 1:** Baseline characteristics of infants in early anemia group and control group (N = 110).

Variable	Early Anemia Group	Control Group	*p*-Value
	N = 55	N = 55	
Maternal factors			
maternal age (year) ^▲^	30 (28–33)	30 (27–35)	0.575
Gravidity ^▲^	2 (1–3)	2 (1–3)	0.593
Parity ^▲^	1 (1–2)	2 (1–2)	0.404
Cesarean section, n (%)	33 (68.8%)	47 (74.6%)	0.528
Singleton, n (%)	24 (50.0%)	44 (69.8%)	0.103
Neonatal factors			
Gestational age (weeks) ^▲^	30.43 (29.29–32.29)	30.43 (29.29–32)	0.657
Birth weight (g) ^▲^	1355 (1190–1420)	1300 (1105–1400)	0.274
Birth head circumference (cm) ^▲^	27 (26–28)	27 (26–28)	0.690
Birth length (cm) ^▲^	38 (37–40)	38 (36–40)	0.579
Male, n (%)	25 (52.1%)	39 (61.9%)	0.336
Nutrition supply			
Amino acid introduction (DOL) ^▲^	0 (0–0)	0 (0–0)	0.258
Duration of amino acid (days) ^▲^	23 (16–34)	21 (14–28)	0.215
Lipid introduction (DOL) ^▲^	1 (1–2)	1 (1–2)	0.791
Duration of lipid (days) ^▲^	21 (15–32)	18 (11–26)	0.076
Start enteral nutrition (DOL) ^▲^	1 (1–2)	1 (1–2)	0.794
Enteral feeding volumes(DOL) ^▲^			
20 mL·kg^−1^·d^−1^	7 (3–12)	5 (4–8)	0.323
50 mL·kg^−1^·d^−1^	12 (7–19)	9 (7–16)	0.187
80 mL·kg^−1^·d^−1^	15 (11–28)	13 (9–22)	0.099
120 mL·kg^−1^·d^−1^	22 (17–36)	18 (13–28)	0.045 *
150 mL·kg^−1^·d^−1^	24 (20–41)	23 (16–33)	0.308
Respiratory condition			
Intubation ventilation (days) ^▲^	2 (0–7)	0 (0–6)	0.340
Non-invasive ventilation, (days) ^▲^	0 (0–4)	2 (0–6)	0.159
Extra oxygen supply, (days) ^▲^	10 (0–23)	5 (0–14)	0.234

DOL, day of life; Data were presented as ^▲^ median (interquartile range) or frequency (percentage). Data were analyzed by the Mann–Whitney U test or Chi-squared test. * *p* < 0.05.

**Table 2:** Comparison of NBNA results between the early anemia group and control group (N = 110).

NBNA Results	Early Anemia Group	Control Group	*p*-Value
	N = 55	N = 55	
NBNA score qualified, n (%)	23 (47.9%)	40 (63.5%)	0.075
NBNA total score ^▲^	36 (35–37.5)	37 (36–38)	0.086
Behavioral ability score ^▲^	10 (9.5–11)	11 (10–12)	0.033 *
Passive muscle tone score ^▲^	8 (8–8)	8 (8–8)	0.818
Active muscle tone score ^▲^	6 (6–7)	6 (6–7)	0.568
Original reflex score ^▲^	6 (6–6)	6 (6–6)	0.282
General assessment score ^▲^	6 (6–6)	6 (6–6)	1.000

NBNA, neonatal behavioral neurological assessments; Data were presented as n(percentage) and ^▲^ median (interquartile range); NBNA score qualified: NBNA total score ≥ 37 considered qualified; Data are analyzed by the Mann–Whitney U test or Chi-squared test. * *p* < 0.05.

**Table 3:** The associations of early anemia with neurodevelopment in very low birth weight preterm infants (N = 110).

NBNA Results	Model 1	*p*	Model 2	*p*	Model 3	*p*
NBNA total score	−0.516 (−1.113–0.082)	0.091	−0.650 (−1.215–−0.085)	0.024 *	−0.748 (−1.368–−0.129)	0.018 *
Behavioral ability score	−0.485 (−0.890–−0.080)	0.019 *	−0.606 (−0.995–−0.218)	0.002 *	−0.457 (−0.840–−0.020)	0.041 *
Passive muscle tone score	−0.036 (−0.197–0.126)	0.666	−0.065 (−0.226–0.096)	0.428	−0.068 (−0.238–0.101)	0.427
Active muscle tone score	−0.205 (−0.726–0.317)	0.442	−0.230 (−0.757–0.296)	0.391	−0.401 (−0.930–0.128)	0.138
Original reflex score	0.847 (−0.054–1.747)	0.065	0.756 (−0.144–1.656)	0.100	0.115 (−0.695–0.925)	0.780
General assessment score	NA	NA	NA	NA	NA	NA

NA: not available. NBNA, neonatal behavioral neurological assessments. Data are presented as β (95% confidence interval). Data were analyzed by a linear model, referred to control group. * *p* < 0.05. Model 1: univariate analysis; Model 2, adjusted for gestational age, biological sex, delivery mode, and birth weight; Model 3, adjusted for the day of enteral feeding volumes reached 120 mL·kg^−1^·d^−1^ based on Model 2.

**Table 4:** Comparison of brain injury between the early anemia group and control group (N = 95).

Brain Injury	Early Anemia Group	Control Group	*p*-Value
	N = 40	N = 55	
Intraventricular hemorrhage	6 (15.0%)	7 (12.7%)	0.489
Ventricular cyst	9 (22.5%)	6 (10.9%)	0.107
Ventricular widening	8 (20.0%)	5 (9.1%)	0.111

Data are presented as n (%). Data were analyzed by Chi-squared test.

## Data Availability

The datasets used during the current study are available from the corresponding author upon reasonable request.
